# Vitamin D_3_ and Dental Caries in Children with Growth Hormone Deficiency

**DOI:** 10.1155/2019/2172137

**Published:** 2019-01-21

**Authors:** Dorota Wójcik, Leszek Szalewski, Elżbieta Pietryka-Michałowska, Janusz Borowicz, Elżbieta Pels, Iwona Beń-Skowronek

**Affiliations:** ^1^Department of Dental Prosthetics, Medical University of Lublin, Poland; ^2^Department of Mathematics and Medical Biostatistics, Medical University of Lublin, Poland; ^3^Chair and Department of Paedodontics, Medical University of Lublin, Poland; ^4^Department of Paediatric Endocrinology and Diabetology with Endocrine-Metabolic Laboratory, Medical University of Lublin, Poland

## Abstract

Vitamin D deficiency is a common risk factor for multifactorial diseases, and it seems to be associated with growth hormone deficiency (GHD). Vitamin D could prevent dental caries. The goal of this study was to identify whether there is an association between hormonal therapy with growth hormone (GH), vitamin D_3_ supplementation, vitamin D_3_ levels, and the occurrence of caries among children affected by GHD. The study group consisted of patients from the Department of Endocrinology and Diabetology of the University Paediatric Hospital at the Medical University of Lublin treated with recombinant human GH for pituitary GHD. It was conducted between October 2014 and June 2015. The study group included 121 children and adolescents aged 6 to 18 years, with 56 children from rural areas and 65 from urban areas. The study group was stratified by the area of residence. We found the statistically significant impact of vitamin D_3_ concentration on the average value of the DMFT (decayed, missed, and filled teeth) index and its component—DT (decayed teeth), which was noted in subjects from rural areas. Among patients from urban areas, we found a statistically significant correlation between duration of therapy and the DMFT index. An increase in duration of GH therapy by 10 months leads to a mean increase in DMFT index by 0.70. Based on multiple regression analysis, we developed the following model: value of DT = 3.10 − 0.73^∗^category of vitamin D_3_ concentration − 0.07^∗^duration of supplementation (in months). In this model, variables with a significant impact on the value of DT in the group of patients from rural areas include time of vitamin D_3_ supplementation and category of vitamin D_3_ concentration. Greater emphasis should be placed on promoting vitamin D_3_ as a potentially effective agent reducing the number of dental caries, especially among patients with GHD.

## 1. Introduction

Vitamin D is a prohormone. It is ingested orally through diet or supplements or produced photochemically in the skin. Vitamin D has diverse biological functions, and its relevance extends beyond bone health and calcium metabolism [1]. Vitamin D plays a crucial role in the maintenance of calcium homeostasis to ensure proper bone mineralization, especially during periods of rapid growth, such as childhood and adolescence. Vitamin D concentration in the body is influenced by season, aging, latitude, physical activity, smoking, or diet [2]. For the last decade, there has been growing interest in vitamin D due to the association between its levels and numerous disorders, such as malignancies, cardiovascular disease, disorders of glucose metabolism, neurodegenerative diseases, and caries [2, 3]. Vitamin D deficiency is a common risk factor for multifactorial diseases [4]. The first reports of the association between vitamin D and teeth cavities appeared in 1928 [5]. Growth hormone deficiency (GHD) is a disorder associated with insufficient excretion of growth hormone (GH). Growth retardation is the main symptom of GHD, manifesting at different times of development in children. GH and insulin-like growth factor (IGF-1) are the two most important factors responsible for the stimulation of growth in children. The prevalence of GHD in a general population ranges from 1/4000 to 1/10,000 people [6, 7]. There may be many causes of congenital GHD—from genetically determined defects of GH synthesis and excretion to organic lesions in the brain. The aetiology of GHD is mostly idiopathic, while genetic abnormalities are responsible for about 5–30% of cases of GHD. Dental caries is a local, extrinsic, pathological process that leads to proteolysis and demineralization of the hard tissues of a tooth. It is a widespread and chronic disease among children, which may cause pain, feeding problems, and sleep disturbances, as well as discomfort and decreased quality of life [8–10].

The most recent research suggests that vitamin D levels influence the function of the GH/IGF-I axis. Vitamin D may increase the production and secretion of IGF-1 (insulin-like growth factor 1) and IGFBP-3 (insulin-like growth factor-binding protein 3) in the liver [11, 12]. On the other hand, both GH and IGF-1 increase the renal production of 1.25-(OH)_2_D (calcitriol) by enhancing the activity of renal 1*α*-hydroxylase; the blood level of vitamin D is also affected [13]. According to the latest results, vitamin D supplementation increases the effect of recombinant human GH on bone formation in children with GHD, which can improve the effects of recombinant human GH therapy [14]. The aim of this study was to identify whether there is an association between hormonal therapy with GH, vitamin D_3_ supplementation, vitamin D_3_ levels, and the occurrence of caries among children affected by GHD.

## 2. Material and Methods

### 2.1. Study Design and Data Collection

The study group consisted of patients from the Department of Endocrinology and Diabetology of the University Paediatric Hospital at the Medical University of Lublin treated with recombinant human GH for pituitary GHD. It was conducted between October 2014 and June 2015. The study group included 121 children and adolescents aged from 6 to 18 years, with 56 children from rural areas and 65 from urban areas. The study group was stratified by the area of residence (Table 1). Children with GHD were divided depending on residence into two comparative groups—children from urban areas and those from rural areas.

In Poland, every child treated with recombinant human GH needs to qualify for a treatment program and may suffer from conditions, such as GHD, Turner syndrome, or chronic renal insufficiency [15]. The inclusion criteria for our study included height standard deviation score (HSDS) ≤ −3, height velocity < 2SD (standard deviation) for age and sex, and maximal GH secretion below 10 ng/mL during stimulation tests (clonidine stimulation test and insulin stimulation test). The exclusion criteria were Turner syndrome, coeliac disease, bone defects, hypothyroidism, renal insufficiency, and neoplasia.

The number of patients undergoing treatment with a recombinant human GH who remained under the care of clinics and who met the study criteria was 302. An estimation of sample size was carried out prior to the research.

The main aim of the study was to identify variables with a statistically significant effect on tooth decay (with particular emphasis on vitamin D_3_ concentration); therefore, a test for one of the *t* correlations was used in order to determine the minimum sample size.

The test showed that for the HO hypothesis, Ro = 0,*α* = 0.05, power = 0.90, and a population correlation of at least Ro = ± 0.30 required*N*(with Fisher corrected = 112). Drawing without replacement was performed from which a 40% sample was taken (121 patients of the clinic).

In 121 patients with pituitary GHD, vitamin D_3_ supplementation was used at a dose of 500–2000 IU/day. Throughout the observation period, patients received recombinant human GH and vitamin D_3_. The duration of vitamin D_3_ supplementation was also recorded. Increased growth rate, IGF-1 concentration within reference values, and progression on the growth chart were indicators of treatment effectiveness. Some of the patients with GHD presented other signs and symptoms of hypopituitarism: secondary hypothyroidism, secondary adrenal insufficiency, or hypogonadotropic hypogonadism treated with the substitution of L-thyroxin, hydrocortisone, and oestradiol in girls or testosterone in boys. The bone age, assessed using the Greulich-Pyle method, was delayed (from 6 months to 3 years) for each child compared to their calendar age.

The preliminary stage consisted of obtaining written consent from the guardians of the children and adolescents participating in the study. The first part of the study was based on a survey containing questions about hygienic and nutritional habits. The second stage consisted of a clinical trial that involved epidemiological dental examinations and laboratory investigations to determine serum levels of vitamin D_3_. Moreover, computed tomography (CT) or magnetic resonance imaging (MRI) scans were acquired to rule out proliferative disorder in accordance with the recommendations of the Polish program for short stature treatment with recombinant human GH in adolescents and children. The guardians were informed of the aims of the study.

The dental assessment was carried out according to the World Health Organisation (WHO) criteria for epidemiological trials [16]. We compared the advancement of caries (mean DMFT index) among patients from different residential areas (urban vs. rural). The study was carried out in agreement with all sanitary requirements using sterile diagnostic kits (i.e., disposable mirrors and probes with artificial lighting). The examiner was assisted by a recording clerk. Results of dental assessment were recorded in standard denture diagrams prepared for each patient. We assessed the severity of dental caries with the use of mean DMFT index, which includes the following components: D—the number of teeth with active decay, M—the number of missed teeth, and F—the number of teeth with fillings.

Blood serum 25(OH)D (vitamin D_3_) levels were measured using the “Elecsys Vitamin D Total” (Roche Diagnostics) chemiluminescence assay (CLIA).

After ruling out proliferative disorders by computed tomography (CT) or magnetic resonance imaging (MRI), qualifying GHD patients received treatment with recombinant human GH. All children received recombinant human GH at a dose of 0.5–1.0 IU per kilogram of body weight a day. Patients presented with normal IGF1 levels and growth rates according to the growth chart appropriate for their sex and age, indicating proper levels of GH in the blood. This study conformed to the STROBE guidelines for reporting observational studies (http://www.strobe-statement.org) [17].

### 2.2. Statistical Analysis

The DMFT index was calculated for each patient and each result was considered a dependent variable. Independent variables included serum levels of vitamin D_3_ and hygienic and nutritional habits of participants. Also, we examined the relationship between independent variables and sex, age, and area of residence, as well as the relationship between dependent variables and classification criteria used in the study. Data were analysed with the Statistica 10 software using Student's *t*-test, Mann–Whitney's *U* test, Spearman's rank correlation coefficient, Pearson's Chi^2^ test, univariate analysis of variance (ANOVA), multiple regression, and Pearson's linear correlation coefficient. A DT value of ≤0.05 was considered statistically significant.

The datasets generated and analysed during the current study are available from the corresponding author on reasonable request.

### 2.3. Ethical Approval

The study was approved by the Medical Ethics Committee of the Medical University of Lublin, Poland.

## 3. Results

No significant impact of vitamin D_3_ concentration on mean DMFT and its components was observed in urban areas. In contrast, a statistically significant impact of vitamin D_3_ concentration on the average value of DMFT index and its component—DT (decayed teeth)—was noted in subjects from rural areas. There was a negative correlation between vitamin D_3_ concentration and the values of mentioned caries indicators (*p* < 0.05).

**Figure 1:**
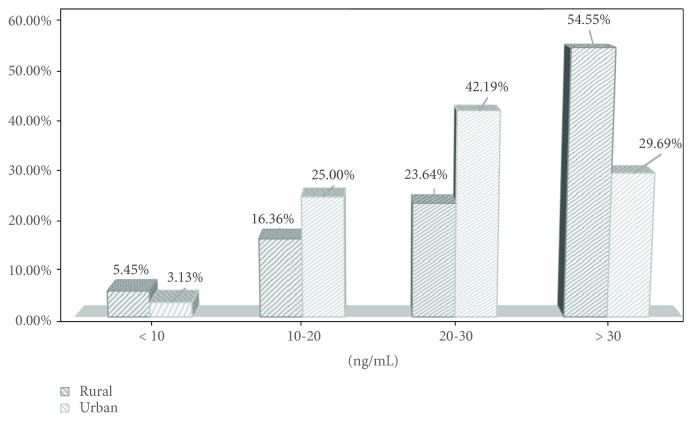
Vitamin D_3_ concentration in four categories in patients' blood serum according to their site of residence.

The mean concentrations of vitamin D_3_ (25(OH)D) did not differ significantly between children from rural areas (29.03 ± 11.23 ng/mL) and children from urban areas (26.17 ± 9.43 ng/mL). Vitamin D_3_ levels were analysed in four categories according to the range of concentrations (0–10 ng/mL—severe deficit, 11–20 ng/mL—deficit, 21–30 ng/mL—suboptimal concentration, and above 30 ng/mL—normal concentration). Vitamin D_3_ concentrations in particular categories (0–10 ng/mL, 11–20 ng/mL, 21–30 ng/mL, and above 30 ng/mL) varied in a statistically significant manner (Pearson's Chi^2^ test = 8.90; df = 3; *p* = 0.031; Figure 1) [18].

In subjects from rural regions, we demonstrated a significant influence of vitamin D_3_ concentration in specific categories on mean DMFT index. Mean DMFT index decreased with an increase in vitamin D_3_ level (categories) (univariate analysis of variance ANOVA *F* = 2.95; *p* = 0.049; Table 2) [18]. On the other hand, in a population of patients from urban areas, there was no statistically significant impact of vitamin D_3_ concentration in the analysed concentration categories on mean DMFT index (univariate analysis of variance ANOVA *F* = 0.44; *p* = 0.73).

There was no significant correlation in both groups (rural and urban) between the duration of therapy with GH and the DT value (test for Pearson's correlation coefficient *r* = −0.07; *t* = −0.48, *p* = 0.63; test for Pearson's correlation coefficient *r* = 0.11, *t* = 0.87; *p* = 0.38). In the rural population, we noted no statistically significant correlation between the duration of therapy and DMFT index (test for Pearson's correlation coefficient *r* = −0.06; *t* = −0.45; *p* = 0.66). Conversely, among patients from urban areas, we found a statistically significant correlation between the duration of therapy and the DMFT index (test for Pearson's correlation coefficient *r* = 0.33; *t* = 2.79; *p* = 0.007).

There is a weak positive correlation. The regression line describing the examined relationships is expressed through the following formula: DMFT = 1.49+ 0.07 ^∗^ duration of therapy (months).

**Figure 2:**
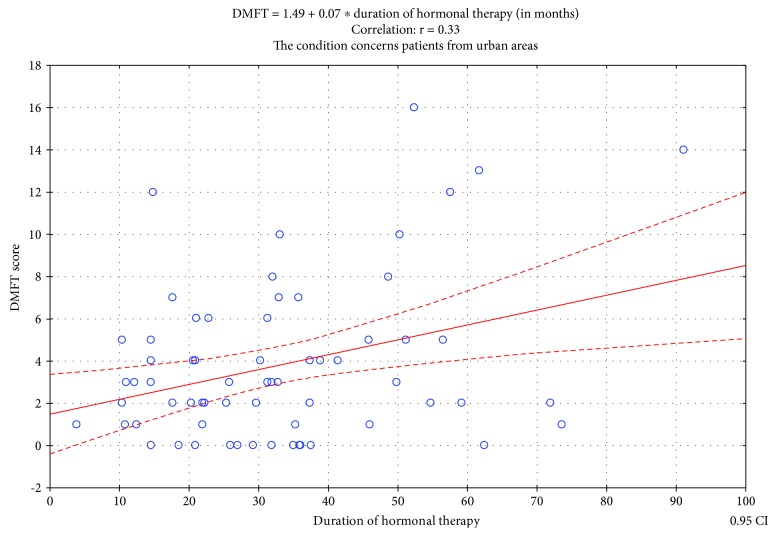
Correlation between duration of hormonal therapy and the DMFT index.

The increase in the duration of GH therapy by 10 units (i.e., 10 months) leads to a mean increase in DMFT index by 0.70 (H0: Ro = 0; power of the test (Fisher's corrected *z* = 0.78; Figure 2)).

There was no statistically significant correlation between duration of hormonal therapy and vitamin D_3_ concentration in subjects from both rural (test for Pearson's correlation coefficient *r* = 0.09; *t* = 0.66; *p* = 0.51) and urban environments (test for Pearson's correlation coefficient *r* = 0.08; *t* = 0.54*p* = 0.59)_._ The duration of vitamin D_3_ supplementation (in months) also has no significant influence on the concentration category in both rural (univariate analysis of variance ANOVA *F* = 0.37; *p* = 0.78), and urban areas (univariate analysis of variance ANOVA *F* = 2.40; *p* = 0.08).

Based on multiple regression analysis, we developed the following model:

(1) $$\begin{array}{c} Value\text{ of }DT=3.10-0.73*category\text{ of }vitamin\;D_{3}\;concentration-0.07*duration\text{ of }supplementation\;\left(\text{in }months\right). \end{array}$$

In this model, variables with a significant impact on the value of DT in the group of patients from rural areas include time of vitamin D_3_ supplementation and category of vitamin D_3_ concentration. An increase in vitamin D_3_ concentration by 1 category results in a mean reduction of DT value by 0.73, while prolonging the vitamin D_3_ supplementation time by 10 months leads to a mean reduction of DT value by 0.70. The developed model for the DT value has an average testing power (multiple regression *F* = 7.28; *p* = 0.002; *R* = 0.46). The power of the multiple regression test equals 0.76; probability level *p* = 0.0018 for *R*^2^ = 0.21and confidence interval for the coefficient of determination assumes values from 0.055 to 0.366.

In the urban environment, we did not find a statistically significant influence of the abovementioned variables on the DT value. Both in urban and rural areas, there was no statistically significant impact of the examined variables on the DMFT index.

We also analysed the influence of seasons of the year on the value of DT and DMFT index, and vitamin D_3_ concentration in rural and urban environments. There was no statistically significant relationship.

## 4. Discussion

Our results showed that higher vitamin D_3_ concentrations and vitamin D supplementation reduces the intensity of active caries in children with GHD. This suggests that caries may constitute a symptom of vitamin D deficiency.

Few authors have described the problem of dental caries among children with GHD. The influence of vitamin D_3_ on the growth and development of caries is well established; thus, we decided to evaluate whether there is a relationship between those two phenomena.

Abnormalities of enamel and dentin in the course of development of hard tooth tissues due to altered vitamin D metabolism may lead to an increased incidence of caries. Numerous studies have reported a correlation between vitamin D concentration in the organism and susceptibility to caries [19, 20]. Schroth et al. observed that preschool children with reduced vitamin and calcium concentrations were significantly more prone to developing dental caries. In their study, children free of caries were twice as likely to have optimal vitamin D (25(OH)D) concentrations (75 nmol/L or 30 ng/mL) than children with severe early childhood caries (S-ECC), who were three times more likely to have vitamin D deficiency (below 35 nmol/L or 14 ng/mL) [8].

The results of Schroth et al. suggest that suboptimal vitamin D concentrations (below 30 ng/mL) are associated with the risk of caries increasing by 39%, while according to the Institute of Medicine (IOM), the lack of optimal vitamin D concentrations (below 20 ng/mL) may elevate the risk of caries development by as much as 47% [21]. Our results indicate that we may observe a statistically significant relationship between vitamin D_3_ concentrations in specific categories on mean DMFT indexes in a rural environment. Mean DMFT indexes decrease as vitamin D_3_ levels (concentration categories) increases.

Kim et al. analysed the relationship between 25(OH)D and the prevalence of caries among Korean children. They took into consideration the data collected from 2003 to 2018 in 1688 children aged 10–12 years. Children with vitamin D_3_ concentrations below 50 nmol/L or 20 ng/mL were exposed to the risk of dental caries in molar teeth by more than 1.295 times compared to children with higher vitamin D_3_ levels [22].

Also, Grant observed an inversely proportional association between exposure to sunlight and the development of dental caries and tooth loss. A meta-analysis by Grant et al. demonstrated that vitamin D concentrations above 30–40 ng/mL reduce the development of caries [23]. Moreover, a meta-analysis by Hujoel (24 controlled clinical trials) assessing the influence of vitamin D supplementation or sun exposure in 2800 patients indicated that vitamin D might prevent development of caries. In 2013, Hujoel published an article, which indicates that additional exposure to sunlight, as well as vitamin D_3_ and D_2_ supplementation were associated with a significant decrease in caries compared to a lack of any kind of vitamin D supplementation [24]. Our results demonstrated a statistically significant relationship between vitamin D supplementation, its concentration in the body, and the DT value. There is a clear impact of the duration of vitamin D_3_ supplementation and the category of vitamin D_3_ concentration on the DT value in the group of patients from rural areas. Elevation of vitamin D_3_ concentration by 1 category (in analysed concentration categories) causes a mean reduction in the DT value by 0.73, while the prolongation of vitamin D_3_ supplementation by 10 months leads to a mean reduction in DT value by 0.79. The developed model for the value of DT is characterised by an average power (multiple regression *F* = 7.28; *p* = 0.002; *R* = 0.4).

The problem of tooth decay among urban and rural children has been frequently touched on by both domestic and foreign authors [10, 25]. Małkiewicz and Kepa studied the prevalence of caries and malocclusion in children from the Masovia region. Their research, similar to ours, showed significant differences between children living in rural and urban areas. The average DMFT index in youths from metropolitan schools was 4.09 and was significantly lower than the values for children from small towns (5.96) and villages (6.88) (*p* < 0.05) [26]. Koc-Gąska and Przybyłek investigated the health status of the oral cavity of 12-year-old children from towns and villages of the Warmia and Mazury region. In their research, the average DMFT index value (showing intensity of caries) was 2.86 with 3.21 for girls and 2.48 for boys. Differences in the DMFT index value, between children living in villages (3.21) and those living in cities (2.77), were also observed [27]. Own research and a statistical analysis that was carried out showed higher values for the DMFT index and for the DT component in rural environments. Significantly lower values of the DT component were observed in the urban population (*p* < 0.04). The average DMFT index was 4.26, while the DT component for rural children was 2.23. For urban children, the average value was 4.12 for the DMFT index and 1.39 for the DT component. According to Koc-Gąska and Przybyłek (as in our research), the prevalence of caries is significantly higher among rural (90%) compared to urban (72%) children [27]. Our research has shown that the occurrence of caries was observed in 91.07% (*n* = 53) of patients from rural environments, whereas the prevalence of caries in the urban population was 81.54% (*n* = 53).

Ciresi et al. examined the prevalence of vitamin D deficiency among Sicilian children with GHD [13]. Ciresi and Giordano also assessed the interrelationships between vitamin D and GH and IGF1, which are well documented, although it is not entirely clear whether GH influences bone and vitamin D metabolism directly or indirectly through increase in IGF1 synthesis. Coadministration of GH and IGF1 leads to elevated vitamin D concentration in healthy individuals. However, some studies indicate that vitamin D concentration does not change significantly with changes in IGF1 and GH levels, as in patients with acromegaly and GHD after treatment [28]. On the other hand, vitamin D seems to influence increased IGF1 production in children and adults [29]. Studies by Ciresi et al. demonstrated frequent vitamin D deficiency among Sicilian children with GH deficiency and an improvement in serum vitamin D levels after 12 months of GH treatment regardless of the season. Ciresi et al. proved that even in countries with high sun exposure, vitamin D deficiency is frequent among children with GHD, especially during winter. Children examined by Ciresi et al. were characterised by average 25(OH)D concentrations of 31.1 ± 11.1 ng/mL during sunny months (from June to August) and average 25(OH)D concentrations of 17.3 ± 5.3 ng/mL during colder months (from November until February) [13]. Delecroix et al. investigated the state of vitamin D (25-hydroxyvitamin D (25(OH)D)) and 1,25-dihydroxyvitamin D (1,25(OH)2D) levels in children treated for GH deficiency due to pituitary stalk interruption syndrome (PSIS). The mean 25(OH)D in examined children amounted to 33.2 ± 18.0 ng/mL. Also, sex and season influenced 25(OH)D levels; concentrations were higher in boys than in girls and during sunny seasons compared to cold ones [30]. Our results failed to demonstrate similar relationships. Witkowska-Sadek et al. examined 84 children and adolescents with GHD. In their study group, the 25(OH)D concentration was 22.3 ± 6.9 ng/mL. In our study, the mean 25(OH)D concentration was 29.03 ± 11.23 ng/mL for children from rural areas and 26.17 ± 9.43 ng/mL for children from urban areas [31]. Also, our results showed that both in rural and in urban environments, there was no statistically significant correlation between the duration of hormonal therapy and vitamin D_3_ concentration.

The lack of a control group is a limitation of our study. However, the examination of 25(OH)D concentrations constitutes the strength of this analysis, as it accurately depicts vitamin D status, taking into consideration vitamin D acquired from food, as well as endogenously. Also, in our study, dental examinations were carried out by trained dentists.

Additional research should investigate the relationship between levels of vitamin D_3_, its supplementation, and the presence of active dental caries in children with GHD. An explanation is necessary as to why some dependencies apply exclusively to rural children, while the duration of GH therapy is correlated with the occurrence of active tooth decay only in urban children. One can assume that there are differences in the lifestyles of rural and urban children suffering from GHD, related primarily to everyday habits influencing oral hygiene, frequency of visits to the dentist, and sun exposure. The amount of sun exposure is probably majorly responsible for serum vitamin D_3_ concentrations in rural children, whereas vitamin D supplementation has a greater influence on those levels in urban children. The duration of therapy with GH and its influence shows a correlation with the incidence of dental caries in urban children suffering from GHD. One can suspect that this is caused by vitamin D_3_ deficiency during the intense growth period influenced by administering the recombinant human GH. Among urban children with GHD, we more often tend to observe concentrations of vitamin D_3_ in the range from 21 to 30 ng/mL, which indicates a vitamin deficiency. This observation is statistically significant.

## 5. Conclusions

1. In rural children who are probably more exposed to sunlight and who are treated for GHD, the number of cavities due to caries decreases with increasing vitamin D_3_ concentrations and the prolongation of vitamin D_3_ supplementation. This indicates that vitamin D_3_ might influence the incidence of cavities in this group of patients
2. Duration of GH therapy in children with GHD from urban areas contributes to an increase in the number of cavities; thus, intensification of dental care, vitamin D_3_ supplementation, and sun exposure should be considered in this group of patients
3. Greater emphasis should be placed on promoting vitamin D_3_ as a potentially effective agent reducing the number of dental caries, especially among patients with GHD

## Figures and Tables

**Table 1:** The study group according to the area of residence.

	Place of residence		Sex		Median age (years)	
	Country	City	Boys	Girls	Country	City
*n*	56	65	92	29	13.45 ± 2.54	13.97 ± 2.26
Total	121		121		13.73 ± 2.40	

**Table 2:** The influence of vitamin D concentration on mean DMFT index and its components in populations of children with GHD from rural areas.

Caries indices	Vitamin D_3_ concentration lower than 10 ng/mL		Vitamin D_3_ concentration lower than 20 ng/mL		Vitamin D_3_ concentration lower than 30 ng/mL		Vitamin D_3_ concentration higher than 30 ng/mL		Kruskal-Wallis test
	Mean	Median	Mean	Median	Mean	Median	Mean	Median	*H* value
DT	4.67 ± 3.73	3.00	3.78 ± 2.68	5.00	1.38 ± 1.27	1.00	1.93 ± 1.66	1.00	7.70
MT	0.00 ± 0.00	0.00	0.00 ± 0.00	0.00	0.00 ± 0.00	0.00	0.00 ± 0.00	0.00	1.88
FT	3.00 ± 2.31	3.00	1.78 ± 1.69	1.00	2.69 ± 2.38	2.00	1.77 ± 1.99	1.00	1.20
DMFT	7.67 ± 2.08	7.00	5.55 ± 2.56	6.00	4.08 ± 2.26	4.00	3.70 ± 3.05	3.50	7.56

DT = decayed teeth; MT = missed teeth; FT = filled teeth; DMFT = decayed, missed, and filled teeth; GHD = growth hormone deficiency.

## Data Availability

The data used to support the findings of this study are available from the corresponding author upon request.
